# Benchmarking natural-language parsers for biological applications using dependency graphs

**DOI:** 10.1186/1471-2105-8-24

**Published:** 2007-01-25

**Authors:** Andrew B Clegg, Adrian J Shepherd

**Affiliations:** 1 School of Crystallography, Birkbeck, University of London, Malet Street, London WC1E 7HX, UK

## Abstract

**Background:**

Interest is growing in the application of syntactic parsers to natural language processing problems in biology, but assessing their performance is difficult because differences in linguistic convention can falsely appear to be errors. We present a method for evaluating their accuracy using an intermediate representation based on dependency graphs, in which the semantic relationships important in most information extraction tasks are closer to the surface. We also demonstrate how this method can be easily tailored to various application-driven criteria.

**Results:**

Using the GENIA corpus as a gold standard, we tested four open-source parsers which have been used in bioinformatics projects. We first present overall performance measures, and test the two leading tools, the Charniak-Lease and Bikel parsers, on subtasks tailored to reflect the requirements of a system for extracting gene expression relationships. These two tools clearly outperform the other parsers in the evaluation, and achieve accuracy levels comparable to or exceeding native dependency parsers on similar tasks in previous biological evaluations.

**Conclusion:**

Evaluating using dependency graphs allows parsers to be tested easily on criteria chosen according to the semantics of particular biological applications, drawing attention to important mistakes and soaking up many insignificant differences that would otherwise be reported as errors. Generating high-accuracy dependency graphs from the output of phrase-structure parsers also provides access to the more detailed syntax trees that are used in several natural-language processing techniques.

## Background

In the last few years, natural language processing (NLP) has become a rapidly-expanding field within bioinformatics, as the literature keeps growing exponentially [1] beyond the ability of human researchers to keep track of, at least without computer assistance. NLP methods have been used successfully to extract various classes of data from biological texts, including protein-protein interactions [2], protein function assignments [3], regulatory networks [4] and gene-disease relationships [5].

Although much headway has been made using text processing methods based on linear pattern matching (e.g. regular expressions), the diversity and complexity of natural language has caused many researchers to integrate more sophisticated parsing methods into their biological NLP pipelines [6,7]. This enables NLP systems to take into account the grammatical content of each sentence, including deeply nested structures, and dependencies between widely separated words or phrases that are hard to capture with superficial patterns.

**Figure 1:**
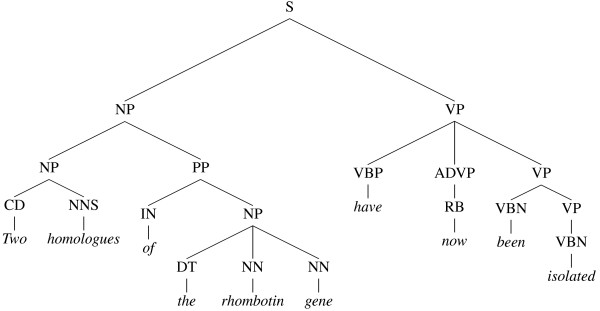
**A constituent (phrase structure) tree**. The phrase structure of the sentence "Two homologues of the rhombotin gene have now been isolated" from the GENIA treebank. The definitions of the linguistic labels used in this and all other diagrams are given in the List of Abbreviations section.

**Figure 2:**
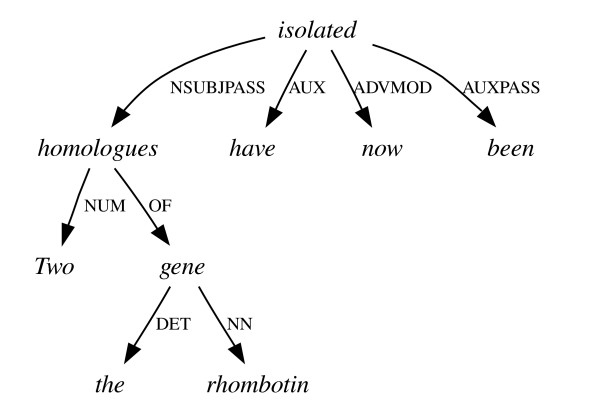
**A dependency graph**. The dependency graph of the sentence in Figure 1.

General-purpose full-sentence parsers fall into two broad categories depending on the formalisms they use to model language and the corresponding outputs they produce. Constituent parsers (or treebank parsers) recursively break the input text down into clauses and phrases, and produce a tree structure where the root represents the sentence as a whole and the leaves represent words (see Figure 1). Dependency parsers model language as a set of relationships between words, and do not make widespread use of concepts like 'phrase' or 'clause'. Instead they produce a graph for each sentence, where each node represents a word, and each arc a grammatical dependency such as that which holds between a verb and its subject (see Figure 2).

While constituent parsers are closer to the theoretical models of language employed in mainstream linguistics, dependency parsers are popular in applied NLP circles because the grammatical relationships that they specify are not entirely unlike the semantic relationships encoding logical predicates to which an NLP developer would like to be able to reduce a sentence. However, there is no such thing as a standard grammar for dependency parsers. Each parser uses a different set of dependency types and a different set of attachment rules, meaning that there is often disagreement between dependency parsers regarding graph topology and arc labels [8,9]. This means that evaluating dependency parsers, and comparing the results of one to another, can be somewhat fraught with complexity.

**Figure 3:**
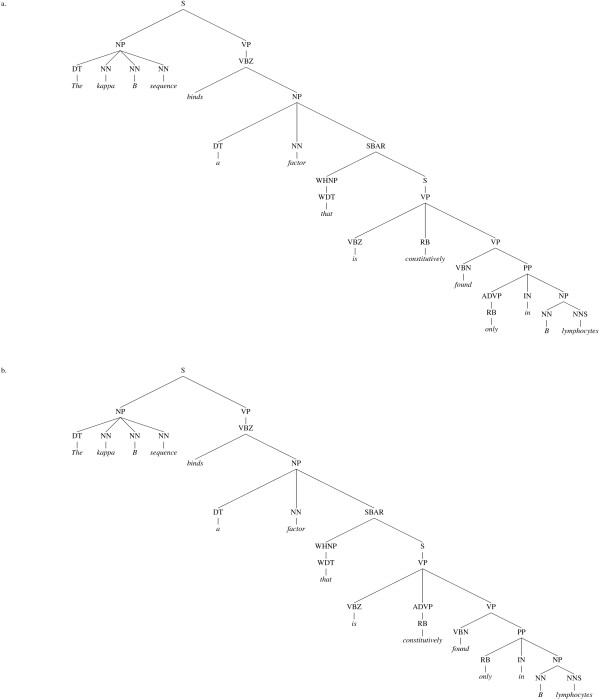
**Adverbial attachment conventions**. In (a), the lexicalised version of the Stanford parser attaches the adverb (RB) "constitutively" via an adverbial phrase (ADVP) to its parent verb phrase (VP). In (b), the unlexicalised Stanford parser skips this step and attaches the adverb directly to the verb phrase. These two representations are semantically equivalent.

**Figure 4:**
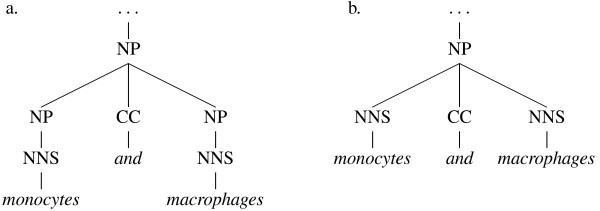
**Co-ordinating conjunction conventions**. Two alternative ways of joining two nouns with a conjunction ("and") – the GENIA corpus uses convention (a), while all of the parsers tested use (b). The additional level of noun phrase (NP) constituents however makes no difference to the meaning.

Due to the impact on computational linguistics of the Penn Treebank (PTB) [10], a vast collection of hand-annotated constituent trees for many thousands of sentences drawn mostly from news reports, there is on the other hand a *de facto *standard for constituent parsers to follow. This means that there are several high-performance parsers available, trained on the PTB, which produce a pre-defined set of clause, phrase and word category (part-of-speech or POS) labels. There are also standardised evaluation measures by which these parsers are benchmarked against a set-aside portion of the original treebank. The most frequently published scores for parser performance use precision and recall measures based on the presence or absence of constituents in each parser's output, compared to the gold standard. These are sometimes referred to as GEIG or PARSEVAL measures, and although their limitiations are well known [11,12] – for example, they have problems distinguishing between genuine errors which would affect the output of NLP applications, and minor differences of convention (see Figures 3 and 4) which would not – they are still in wide use.

The impact of the PTB is also such that both the major linguistic annotation projects for molecular biology corpora [13,14] employ largely PTB-like conventions, although the amount of annotated biological text is currently at least an order of magnitude less than that which is available in the general-English domain. Although the quantities available are insufficient for retraining parsers, evaluation of the performance of parsers for bioinformatics applications is possible given a meaningful evaluation technique.

**Figure 5:**
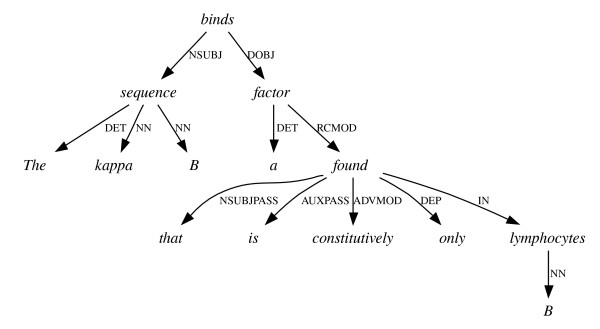
**Adverbial attachment using dependencies**. Either of the representations in Figure 3 result in this graph.

**Figure 6:**
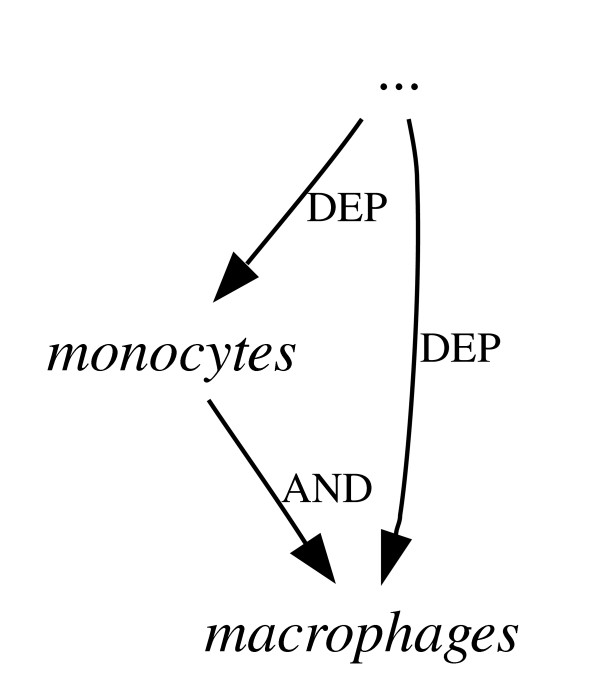
**Co-ordinating conjunctions using dependencies**. Either of the representations in Figure 4 result in this graph fragment.

Although a dependency graph for a sentence will not, typically, contain as much information as a constituent tree for the same sentence, it is possible to transform the tree structure into a dependency graph by employing a set of deterministic mapping functions [9]. The mapping procedure often results in the elimination of redundant information found in the tree structure, and thus tends to level out many of the insignificant differences in convention between alternative constituent parses (see Figures 5 and 6).

This process therefore provides a convenient way to evaluate constituent parsers on those aspects of their output that most affect meaning, as well as forming a useful intermediate representation between phrase structure and logical predicates. Furthermore, given such a framework, it becomes easy to define application-specific evaluation criteria reflecting the requirements that will be placed upon a parser in a biological NLP scenario. Using this approach, we have evaluated several leading open-source parsers on general syntactic accuracy, as well as their ability to extract dependencies important to correct interpretation of a corpus of texts relating to biomolecular interactions in humans. The parsers are scored on their ability to correctly generate the grammatical dependencies in each sentence, by comparing the corresponding dependency graphs from their output and from the constituent structure of the original treebank. The results are presented below.

## Results and Discussion

The software packages used in our evaluation are the Bikel parser [15], the Collins parser [16], the Stanford parser [17,18] and the Charniak parser [19] – including a modified version known herein as the Charniak-Lease parser [20]. All of these are widely used by the computational linguistics community, and have been employed to parse molecular biology data (see Related Work section), despite having been developed and trained on sentences from the Penn Treebank. While it may be the case that, over the coming years, enough consistently-annotated biological treebank data becomes available to make retraining parsers on biological text a feasible proposition, this is by no means true yet. Furthermore, when choosing which parser to retrain with such data as and when it becomes available, one would wish to pick one which had already demonstrated good cross-domain portability, since the biomedical domain in fact encompasses multiple subdomains with distinct sublanguages [21].

We tested at least two versions of each parser as it is by no means certain *a priori *that the best-performing version on the PTB will likewise perform best on biological text. Our gold standard corpus was 1757 sentences from the GENIA treebank [13], which were mapped from their original tree structures to dependency graphs by the same deterministic algorithm from the Stanford toolkit that we used to convert the output of each parser [9]. See the Methods section for more details of our parsing pipeline.

### Overall parse accuracy

For each parser, we calculated two scores, constituent effectiveness (F_*const*_) and dependency effectiveness (F_*dep*_)against the original constituent trees in the treebank, and their dependency graph equivalents, respectively (see Tables 1 and 2). These scores are measures of tree or graph similarity between the parser output and the gold standard corpus, penalising false negatives and false positives – see the Methods section for the formulae used to calculate them. When comparing the parsers' output in terms of dependency graphs rather than raw trees – that is, using F_*dep *_rather than F_*const *_– there is a much less gradual spread, with the three front-runners being clearly separated from the rest.

**Table 1:** F_*const *_score, all sentences

Parser	F_*const*_
Charniak-Lease	80.2
Bikel (0.9.8)	79.4
Bikel (0.9.9c)	79.4
Charniak (Aug 05)	78.1
Collins (model 2)	77.8
Collins (model 3)	77.2
Collins (model 1)	76.4
Stanford (unlexicalised)	72.3
Stanford (lexicalised)	71.1

Parser effectiveness based on comparison of constituent trees to the GENIA treebank, summed over entire corpus.

**Table 2:** F_*dep *_score, all sentences

Parser	F_*dep*_
Charniak-Lease	77.0
Bikel (0.9.8)	77.0
Bikel (0.9.9c)	77.0
Stanford (lexicalised)	70.5
Charniak (Aug 05)	68.5
Stanford (unlexicalised)	68.5
Collins (model 2)	68.0
Collins (model 1)	68.0
Collins (model 3)	67.0

Parser effectiveness based on comparison of dependency graphs to the graphs generated from the GENIA treebank, summed over the entire corpus.

Note that the F_*dep *_scores given in Table 2 use the strictest criterion for a match between a dependency in the parse and the corresponding dependency in the gold standard. A match is only recorded if an arc with the same start node, end node and label (dependency type) exists. This is important as the type of a dependency can be crucial for correct interpretation, discriminating for example between the subject and direct object of a verb. However, many assessments of dependency parsers use a weaker matching criterion which disregards the dependency type, and thus only takes into account the topology of the graph and not the arc labels. For comparison purposes, the mean scores using this weaker untyped criterion are given in Table 3 (see also the Related Work section). Note that the rank order of the parsers is the same when using the less strict matching criterion, apart from some slippage by the lexicalised version of the Stanford parser, suggesting that this parser's scores on the strict test are boosted by comparatively good dependency type identification. All scores in this paper use the strict matching criterion unless otherwise specified.

**Table 3:** F_*dep *_score, all sentences, loose matching criterion

Parser	F_*dep*_
Charniak-Lease	81.0
Bikel (0.9.8)	81.0
Bikel (0.9.9c)	81.0
Charniak (Aug 05)	78.0
Stanford (unlexicalised)	74.5
Collins (model 2)	72.5
Collins (model 1)	72.5
Stanford (lexicalised)	72.5
Collins (model 3)	71.5

Parser effectiveness based on comparison of dependency graphs to the graphs generated from the GENIA treebank, summed over the entire corpus, disregarding dependency types.

The overall effectiveness scores for some of the parsers are distorted, however, by the fact that they encountered sentences which could not be parsed at all (Table 4). It is useful to separate out the effects on the mean scores of complete parse failures as opposed to individual errors in successfully-parsed sentences. The F_*dep *_scores in Table 5 show the mean effectiveness for each parser averaged *only *over those sentences which resulted in a successful parse. The Bikel parser version 0.9.9c claims in its release notes that the parser has a new robustness feature meaning that it "should *always *produce *some *kind of a parse for every input sentence" (original emphasis) [22], but this does not appear to be true for biological texts. However, it is an improvement over version 0.9.9 (not featured in this investigation) which we found to suffer from 440 failures (25% of the corpus) on the GENIA treebank [12]. The parse failures for all of the parsers tended to occur in longer, more complex sentences.

**Table 4:** Parse failures

Parser	Failures
Charniak-Lease	0
Charniak (Aug 05)	0
Stanford (unlexicalised)	0
Stanford (lexicalised)	0
Bikel (0.9.8)	1
Bikel (0.9.9c)	2
Collins (model 1)	12
Collins (model 2)	25
Collins (model 3)	40

Number of sentences which completely failed to parse, out of a total of 1757 in the whole corpus.

**Table 5:** F_*dep *_score, successfully parsed sentences only

Parser	F_*dep*_
Charniak-Lease	77.0
Bikel (0.9.8)	77.0
Bikel (0.9.9c)	77.0
Collins (model 3)	71.0
Collins (model 2)	70.5
Stanford (lexicalised)	70.5
Collins (model 1)	69.5
Charniak (Aug 05)	68.5
Stanford (unlexicalised)	68.5

Parser effectiveness based on comparison of dependency graphs to the graphs generated from the GENIA treebank, summed over the sentences for which each parser returned a successful parse.

The highest-scoring parsers overall, the Charniak-Lease parser and the Bikel parser, achieve very similar scores. Therefore, we decided to subject these two parsers to a series of tests designed to determine where the strengths and weaknesses of each lay when assessed on tasks important to biological language processing applications. We used the older version of the Bikel parser (0.9.8) as it suffered only one failure, as opposed to two by version 0.9.9c.

### Prepositional phrase attachment

One problem that is frequently cited as hard for parsers is the correct attachment of prepositional phrases – modifiers attached to nouns or verbs that convey additional information regarding time, duration, location, manner, cause and so on. It is important to correctly attach such modifiers as errors can alter the meaning of a sentence considerably. For example, consider the phrase "Induction of NF-KB during monocyte differentiation by HIV type 1 infection." Is it the induction (correct) or the differentiation (incorrect) which is caused by the infection? Furthermore, the targets of many biological interactions are expressed in prepositional phrases, e.g. "*X *binds **to *Y***" – the bold section is a prepositional phrase. However this problem is non-trivial because correct attachment relies on the use of background knowledge (for humans), or an approximation of background knowledge based on frequencies of particular words in particular positions in the training corpus (for parsers). These frequencies are often sparse, and for previously unseen words (e.g. many of the technical terms in biology) they will be missing altogether.

To assess the potential impact of this phenomenon, we tested the two best parsers on their ability to correctly generate dependencies between prepositions and both the head words of the phrases they modify and the head words of the modifying phrases, by calculating F_*dep *_scores over just these arcs. (We did not penalise the Bikel parser for missing dependencies in the one sentence it failed to parse at all, in any of these tasks.) For example, in the phrase "inducing NF-KB expression in the nuclei," the modifying phrase of the preposition "in" is "the nuclei" – "nuclei" being the head of this phrase – and the modified word is "inducing". The results are given in Table 6. Surprisingly, both parsers scored slightly higher on the harder portion of this task (attaching prepositions to the appropriate modified words) than they did across all dependency types, where both achieved an F_*dep *_of 77.0 as shown in Table 5. On the easier portion of this task (attaching prepositions to the appropriate modifying words), both scored considerably higher. This ran contrary to our expectations, and indicates that the conventional 'folk wisdom' that prepositional phrase attachment is a particularly hard task is not necessarily true within the constrained environment of biological texts.

**Table 6:** F_*dep *_for prepositional phrase attachment

Parser	Modified words F_*dep*_	Modifying words F_*dep*_
Charniak-Lease	79.5	89.5
Bikel (0.9.8)	78.0	91.0

Parser effectiveness for the task of attaching prepositions correctly to the words they modify and to the words which are doing the modifying.

### Reconstructing co-ordinating conjunctions

**Figure 7:**
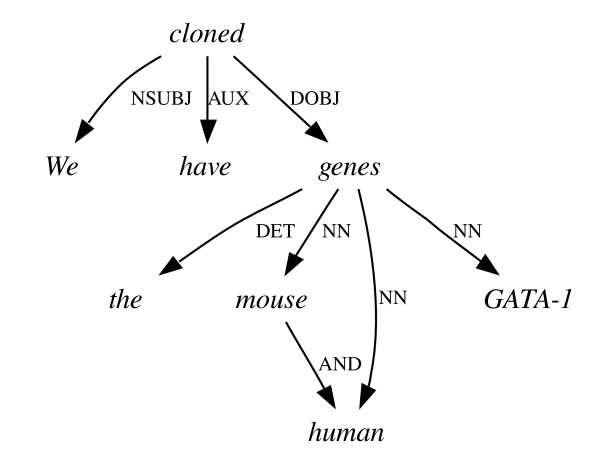
**Co-ordination ambiguity I**. The correct dependency graph for the sentence "We have cloned the mouse and human GATA-1 genes."

**Figure 8:**
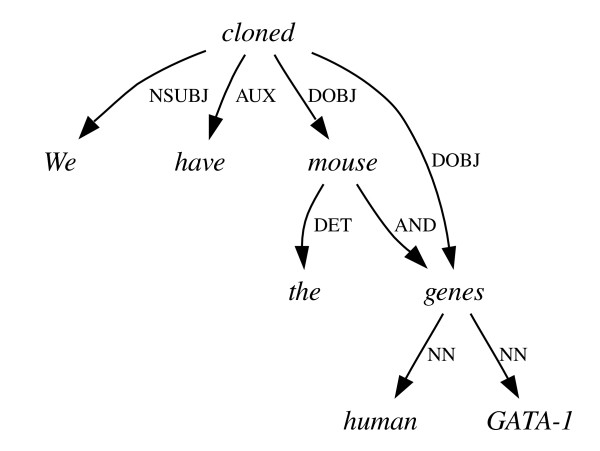
**Co-ordination ambiguity II**. An incorrect graph for the sentence in Figure 7, implying that some genes and a mouse have been cloned.

Another syntactic phenomenon that is problematic for similar reasons is co-ordinating conjunction – the joining on an equal footing of two equivalent grammatical units (e.g. two noun phrases) by a conjunction such as 'and' or 'or'. Since the scope of the conjunction relies on extra-linguistic knowledge or assumptions, there are often several equally grammatical but semantically quite different readings available. An example of this is given in Figures 7 and 8. The correct reading (Figure 7) refers to the cloning of GATA-1 genes from mice and humans – "mouse" and "human" are both attached directly to "genes". An alternative, grammatical, yet incorrect reading is shown in Figure 8, where "human" is attached to "genes", but "mouse" is attached directly to "cloned", implying that some human genes and a whole mouse were cloned.

To measure the ability of the parsers to make the right choices in these situations, we recalculated the F_*dep *_score over only those subgraphs (in the parse or the gold standard) whose root words are at either end of a conjunction dependency. For example, if we were comparing the incorrect parse in Figure 8 to the sentence in Figure 7, our gold standard would consist of all the dependencies from Figure 7 that go to or from the words "mouse" and "human", as these are connected by the conjunction AND. Our test set would consist of all the dependencies in Figure 8 that connect to any of the words "the", "mouse", "human", "GATA-1" and "genes", as the conjunction joins the words "mouse" and "genes" upon which the words "the", "human" and "GATA-1" depend. True and false positive counts, and thus precision, recall and F_*dep *_(see Methods section) can then be calculated over just these dependencies. It would not be sufficient to compare the conjunction dependency alone between the two graphs as this would not measure the extent of this initial error's consequences. In some circumstances, such as nested co-ordinations involving complex multiword phrases – e.g. "the octamer site and the Y, X1 and X2 boxes" – these consequences can be particularly far-reaching. Both parsers' scores on this task (Table 7) were slightly lower than their averages of 77.0 across all dependency types, but not spectacularly lower.

**Table 7:** F_*dep *_for co-ordinating conjunctions

Parser	F_*dep*_
Bikel (0.9.8)	75.5
Charniak-Lease	75.0

Parser effectiveness for the task of reconstructing phrases joined by conjunctions such as 'and' and 'or'.

### Detecting negation

Reliably distinguishing between positive and negative assertions and determining the scope of negation markers are perennial difficulties in NLP, and have been well studied in the medical informatics context [23,24]. It is not uncommon in information extraction projects to skip sentences containing negation words [4], but 'not' appears in 10% of the sentences in our test corpus, and this figure does not count all the other ways of negating a statement in English. Thus a case should be made for attempting to tackle the problem in a more methodical way. In order to gain some initial insight into whether dependency parses might be of use here, we calculated the F_*dep *_score for all dependency arcs beginning or ending at any of these words: 'not', 'n't', 'no', 'none', 'negative', 'without', 'absence', 'cannot', 'fail', 'failure', 'never', 'without', 'unlikely', 'exclude', 'disprove', 'insignificant'. The results (Table 8) are encouraging and the use of dependency graphs in resolving negations warrants further investigation. The difference between these two parsers is much clearer in this task than in any of the others, and demonstrates that the Charniak-Lease parser may be particularly suited to tackling this problem, as it scores higher than its all-dependencies average while the Bikel parser scores considerably lower.

**Table 8:** F_*dep *_for negation words

Parser	F_*dep*_
Charniak-Lease	80.5
Bikel (0.9.8)	70.5

Parser effectiveness for the task of attaching negation words correctly.

### Verb argument assignment

Although there are uncountably many ways to express most logical predicates in natural language, molecular biology texts and abstracts in particular are generally rather constrained and essentially designed for the efficient reporting of sequences of facts, observations and inferences. As a result, much of the important semantic content in this genre is encoded in the form of declarative statements, where a main verb expresses a single predicate more or less exactly, and its syntactic arguments (the subject, direct object and any indirect or prepositional objects) correspond to the entities over which the predicate holds. This being the case, it is important that the arguments of content-bearing verbs are assigned correctly. Failing to recover the subject or object of a verb will render it less useful – not completely useless, however, since we may like to know e.g. that "*X *inhibits B cell Ig secretion" even if we do not yet know what *X *is. Furthermore, most biologically-important predicates are very much directional, meaning that a confusion between subject and object at the level of syntax will lead to a disastrous reversal of the roles of agent and target at the level of semantics. Put more simply, "*X *phosphorylates *Y*" and "*Y *phosphorylates *X*" are very different statements.

In order to detect any latent parsing problems that might hinder this process, we chose one of the most common biological predicate verbs in the corpus ('induce' in any of its forms) and divided the dependency types that can hold between it and its (non-prepositional) arguments into two sets: those which one would expect to find linking it to its agent, and those which one would expect to find linking it to its target. For example, in the statement "Cortivazol significantly induced GR mRNA," 'Cortivazol' is the agent and 'GR mRNA' is the target. We then calculated an F_*dep *_score for each parser over these dependencies only, counting as a match those which connect the correct two nodes and which are from the correct set, even if the exact dependency type is different. For example, if the gold standard contained a NOMINAL_SUBJECT dependency between two nodes, and the parse contained a CLAUSAL_SUBJECT dependency between the same two nodes, this would count as a match since both are in the agent dependencies set.

The resulting F_*dep *_scores are given in Table 9, together with a breakdown of false negatives (recall errors): the numbers of mismatches (substitutions for dependencies from the other set), non-matches (substitutions for dependencies from neither set), and completely missing dependencies. The scores for both parsers are very high, with the Charniak-Lease parser only mis-categorising one out of 145 instances of arguments for 'induce' (putting it in the wrong category) and proposing only three other erroneous arguments for this verb in the whole corpus. These results bode well for the semantic accuracy of information extraction systems based on these principles.

**Table 9:** Verb argument assignment for 'induce'

Parser	*F*_*dep*_	False posititives	False negatives	Mismatches	Nonmatches	Missing
Charniak-Lease	98.0	4	1	1	0	0
Bikel (0.9.8)	97.0	4	3	1	0	2

Parser effectiveness at assigning the arguments of the verb 'induce' into the correct category (agent or target). For each recall error (false negative), we counted the number of mismatches (substitutions for dependencies from the other set), non-matches (substitutions for dependencies from neither set), and completely missing dependencies.

### Error analysis

Our previous experiences with parser evaluation have indicated the importance of correct POS tagging for accurate parsing; this is demonstrated by the difference in performance between the Charniak-Lease parser, and the other – newer – version of the Charniak parser which does not have the benefit of biomedical-domain POS tagging. To measure the consequences of POS errors, we counted the number of false negatives (recall errors) in the outputs of our two leading parsers where either one or two of the words which should have been joined by the missing dependency were incorrectly tagged. (Remember that, since the strict matching criterion is being applied here, a recall error means that a dependency *of a specific type *is missing; it will often be the case that another dependency of a different type has been substituted.)

Also, in a very small minority of cases, it is possible for nodes to be present in a dependency graph from the gold standard, but actually missing from the same graph in a parser's output, or *vice versa*. This comes about since punctuation symbols are not always retained as nodes in the graph in the same way that words are. If a word is mistakenly treated as a discardable punctuation symbol, it will be omitted from the dependency graph. This can come about as a result of a POS tagging error, an error in the Stanford algorithm or a mismatch between the conventions used by a parser or the gold standard and those used by the Stanford algorithm's developers. Conversely, if a punctuation symbol is treated as a word for the same reasons, it may be present as a node in its own right in the resulting graph even if it would otherwise have been suppressed. Therefore, we also counted the number of missing dependencies in each parser's output where one or both of the nodes that the dependency should have connected were also missing. The results of both of these tests are given in Table 10. The results – one in five missing dependencies being associated with at least one POS error for the Charniak-Lease parser, and almost one in three for the Bikel parser – should provide all the more motivation for the development and refinement of biological POS tagging software.

**Table 10:** Reasons for recall errors

Parser	1 bad tag	2 bad tags	1 missing	2 missing
Charniak-Lease	20.6%	2.0%	0.4%	0.0%
Bikel (0.9.8)	28.6%	3.4%	0.4%	0.0%

For each dependency from the gold standard that was not generated by each parser, we determined whether one or both of the words it should have joined were badly POS-tagged, or entirely missing from the dependency graph of the parser's output.

In addition, we counted the missing dependencies for each parser by type, in order to get an idea of which types were the most problematic. The results (Table 11) are rather interesting. The same five types (out of roughly 50) account for the majority of errors in both cases, although there is some difference in the relative proportions. One in five missing dependencies are of the generic DEPENDENT type, which the Stanford algorithm produces when it cannot match a syntactic construction in a phrase structure tree to a more specific type of dependency. The presence of large numbers of DEPENDENT arcs in the graphs of the gold standard corpus indicates that the GENIA annotators are using syntactic constructions that are unfamiliar to the Stanford algorithm. On closer inspection, we discovered that one fifth of the DEPENDENT arcs missed by each parser had been substituted for more specific dependencies joining the same words; it is impossible for us to judge by comparison to GENIA whether the types of these dependencies are truly correct or not.

**Table 11:** Recall errors by type (top five types only)

Bikel (0.9.8)		Charniak-Lease	
DEPENDENT	20.8%	DEPENDENT	20.4%
PREPOSITIONAL_MODIFIER	12.4%	NOUN_COMPOUND_MODIFIER	11.7%
PUNCTUATION	11.6%	PREPOSITIONAL_MODIFIER	11.6%
ADJECTIVAL_MODIFIER	8.2%	PUNCTUATION	10.5%
NOUN_COMPOUND_MODIFIER	8.0%	ADJECTIVAL_MODIFIER	7.0%

For each parser, these are the five most common dependency types that were not correctly generated, with the proportion of all recall errors they account for.

### Computational efficiency

Full syntactic parsing is a computationally demanding process, and although it is trivial to parallelise by parsing separate sentences on separate CPUs, processing speed is nevertheless an important consideration. We measured the parsing time of the 1757-sentence corpus using the GNU time utility, calculating the total processor time for each parser as the sum of the user and system times for the process. The Charniak-Lease parser took 1 h:18 m:36 s while the Bikel parser took much longer at 7 h:21 m:08 s. These times do not include pre- or post-processing scripts, or the time required to generate the dependency graphs, although these are minor compared to the actual parsing process. All processes were running on one processor of a 3 GHz SMP Linux PC.

The difference between these two results is startling. The Bikel parser is written in Java and the Charniak parser in C++, but this in itself does not explain the difference. Analysis of the time command's output indicated that the Bikel parser had vastly greater memory requirements, and while the Charniak-Lease parser ran without needing to swap any of its data out to the hard disk, the Bikel parser made very frequent use of the swapfile. The newer version of the Bikel parser, while not quite as robust, made a time saving of over 50% compared to its predecessor, which indicates that comptutational speedups are possible and practical with Bikel's architecture. The other parsers in the evaluation varied hugely, ranging from slightly under an hour (for the model 1 Collins parser) to nearly 10 hours (for the lexicalised Stanford parser).

## Conclusion

We have presented a method for evaluating treebank parsers based on dependency graphs that is particularly suitable for analysing their capabilities with respect to semantically-important tasks crucial to biological information extraction systems. Applying this method to various versions of four popular, open-source parsers that have been deployed in the bioinformatics domain has produced some interesting and occasionally surprising results relevant to previous and future NLP projects in this domain.

In terms of overall parse accuracy, the Charniak-Lease parser – a version of the venerable Charniak parser enhanced with access to a biomedical vocabulary for POS-tagging purposes – and version 0.9.8 of the Bikel parser achieved joint highest results. Both parsers relied on good POS tagging to achieve their scores, with large proportions of the dependency recall errors being attributable to POS errors. An interesting comparison can be drawn here between the Charniak-Lease parser, for which just over 20% of the missing dependencies connect to at least one incorrectly-tagged word, and the original Charniak parser, which uses a POS-tagging component trained on newspaper English, and for which almost 60% of the recall errors relate to at least one incorrectly-tagged word.

Both parsers performed well on tasks simulating the semantic requirements of a real-world NLP project based on dependency graph analysis, and achieved mostly similar scores. The reconstruction of co-ordinating conjunctions (e.g. 'and'/'or' constructs) was slightly more difficult than average for each parser, and the correct attachment of negation words (e.g. 'not' or 'without') proved problematic for the Bikel parser, although the Charniak-Lease parser was more successful on this task. Both parsers identified the arguments of the verb 'induce' almost perfectly when we relaxed the matching criterion to allow substitutions between agent-argument dependencies (e.g. NOMINAL_SUBJECT and CLAUSAL_SUBJECT) and between target-argument dependencies (e.g. DIRECT_OBJECT and INDIRECT_OBJECT).

### Practical considerations

There are two additional criteria upon which one might choose a parser for an information extraction project, all other things being equal: robustness and computational efficiency. On the former criterion, the Charniak-Lease parser is slightly more desirable, as it did not fail to parse any of the sentences in the corpus, whereas version 0.9.8 of the Bikel parser failed on one sentence. This seems to reflect an architectural difference between the two parsers; the version of the Charniak parser tested here did not suffer any failures either, and neither did two previous versions that we tested in earlier experiments, whereas the later version of the Bikel parser tested here failed twice (and was itself a bugfix release for a version that failed a staggering 440 times on our corpus). In terms of efficiency, the Charniak parser family is the clear winner, with the Charniak-Lease parser taking a fraction of the time of the Bikel parser to produce slightly better results.

### Advantages of dependency graphs

Given that none of the parsers in this evaluation use dependency grammars natively, one might ask two questions. Firstly, what are the practical advantages of translating the output of treebank-style constituent parsers into dependency graphs? And secondly, how do the graphs thus generated compare to the raw output of dependency parsers on biological texts? We will address the latter question below in the Related Work section. In answer to the former question, the benefits are manifold and apply to both the evaluation process and the engineering of NLP applications.

We hope that the semantic evaluation tasks presented in this paper demonstrate the ease by which application-specific benchmarks can be designed and applied with reference to dependency graphs. Granted, one could conceive of similar phrase-structure tree-based algorithms to test the positioning of, say, negation words with respect to the words they modify, but these would require the comparison of two subtrees and would therefore require much more coding and processing than their dependency equivalents. Indeed, since several subtrees can result in the same grammatical relation (e.g. Figures 3 and 4), one would have to manually account for a degree of allowable variation.

Furthermore, some application-specific tests – such as the analysis of arguments for the verb 'induce' in our investigation – would be impossible using raw constituent trees. This kind of information is not explicitly represented in constituent trees, but rather is implicit (albeit buried rather deeply) in the phrase structures and the rules of English, and to test such relations from trees alone requires the design and implementation of mapping rules that would essentially result in dependency structures anyway.

That said, there is more information in a constituent tree than in its dependency equivalent, and there are many algorithms that make use of the richness of trees in order to tackle such problems as pronoun resolution [25], labelling phrases with semantic roles such as CAUSE, EXPERIENCER, RESULT or INSTRUMENT [26], automatic document summarisation [27], unsupervised lexicon acquisition [28], and the assignment of functional category tags like TEMPORAL, MANNER, LOCATION or PURPOSE to phrases [29]. All of these features may be of use in a fully-featured NLP system, so it is desirable to retain the original phrase-structure representation of each sentence as well as the final dependency graph. Therefore, a parsing pipeline that produces both a constituent tree and a dependency graph has an advantage over one that produces only one of these.

### Related work

The inspiration for this paper came from the observation that constituent parsers are beginning to appear in bioinformatics papers on a wide variety of topics, but without any analysis of how well they perform as isolated components in broader projects. For example, the Bikel parser has been used to produce rough treebanks for human correction in a biological treebanking initiative [30]. Subtrees from the Collins parser have been used as features in a protein interaction extractor [2] and in a classifier for semantic relations between biomedical phrases [31]. The Charniak parser has been employed to assist in the re-ranking of search results in a search engine for genomics documents [32] and in the acquisition of causal chains from texts about protein interactions [33].

The Stanford parser has been used to provide syntactic clues for identifying key clinical terms in the medical domain [34] and gene and protein names in the biological domain [35], although we disagree with the latter paper that unlexicalised parsers – those that represent words simply by their POS tags – are more suited to the biological domain than lexicalised parsers equipped with a general-English lexicon. While the relative positions of the lexicalised and unlexicalised versions of the Stanford parser in our study depend on which evaluation measure is used, both versions were consistently out-performed by the Bikel and Charniak-Lease parsers, both of whose parsing engines are lexicalised with a general-English vocabulary.

A thorough analysis of the effectiveness of these parsers in this domain is vital to identifying the source of errors, to developing workarounds for these errors, and indeed to selecting the right parser to begin with. The work reported here builds on a previous paper on the same subject [12] but the dependency-based approach circumvents many of the limitations of constituent-based evaluation that were identified in the course of that investigation. However, there have been a few papers that deal with the benchmarking of parsers of various kinds on biological or biomedical tasks. Lease and Charniak [20], in introducing the modified version of the Charniak parser that performed so well here, present some comparative scores for various versions of the parser on both the GENIA treebank and the Penn Treebank, but they use constituent-based precision, recall and F-measure (F_*const*_) and therefore implicitly suffer from the inability of such measures to distinguish between differences of meaning and convention (as discussed above in the Background section).

Grover *et al*. [36] present several experiments on parsing MEDLINE abstracts with three hand-crafted grammars. First they demonstrate that although the low-coverage but high-accuracy ANLT parser [37] can return a successful parse on only 39.5% of the sentences in their 79-sentence test set, 77.2% of those sentences (30.5% overall) were parsed perfectly. This strategy seems somewhat dubious for real-world applications, however, since a parse with a handful of minor errors is surely more desirable in practice than no parse at all. The ANLT parser also returns a set of logical predicates representing the sentence; whether this is more or less useful for application development than a dependency graph remains to be seen. They then present some experiments on using the Cass [38] and TSG [39] parsers to correctly interpret compound nouns which encode predicate relationships, differentiating for example between 'treatment response' = response TO treatment, and 'aerosol administration' = administration BY aerosol. Their results for this unique investigation are interesting and encouraging, but it is unfortunate that they do not apply the ANLT parser to the compound noun task, and conversely, they do not provide general measures of coverage and accuracy for the Cass and TSG parsers.

Other papers have been published on the behaviour of native dependency parsers on biomedical text. The paper by Pyysalo *et al*. [8] is perhaps the closest to our own work. They compare the free Link Grammar parser [40] to a commercial parser, the Connexor Machinese Syntax parser [41], both of which have been used in bioinformatics [7,42]. The parsers use different dependency grammars, so the authors prepared a 300-sentence protein-protein interaction corpus with a dual annotation scheme that accommodated the major differences between the two parsers' dependency types. They also disregarded dependency types, as well as directions, as the Link parser's 'links' are not explicitly directional, resulting in an even looser matching criterion than the loose criterion mentioned in our Results section.

The Link parser can return multiple parses in ranked order of likelihood, and taking only the first parse for each sentence, it achieved a recall of 72.9%, and parsed 7.0% of sentences perfectly, although the same group shows elsewhere [43] that this figure may be raised slightly by using an independently-trained re-ranker. The Connexor parser returns a single parse for each sentence; it scored 80.0% for recall and also achieved 7.0% perfect parses. For comparison, our best parser (Charniak-Lease) achieved an overall recall of 81.0% and parsed an impressive 23.1% of sentences perfectly, even given a slightly stricter dependency matching criterion. The authors also scored the parsers on their ability to return perfect interaction subgraphs – minimal subgraphs joining two protein names and the word or phrase stating their interaction – although we disagree that a *perfect *interaction subgraph is necessarily a pre-requisite for successful retrieval of an actual interaction. (Neither is it sufficient, since a negation word might be outside the interaction subgraph yet still able to completely reverse its meaning.)

Schneider *et al*. [44] present results comparable to ours for the Pro3Gres parser [45] on performing several specific syntactic tasks over a small subset of GENIA. Their general approach is very similar to ours, but they do not provide performance indicators over all dependency types, and they chunk multi-word terms into single elements before parsing. They report F_*dep *_scores of 88.5 and 92.0 for identifying the subjects and objects of verbs respectively, although it is not clear whether or not these relation types are defined as broadly as the categories we used above in the study of the verb 'induce', where the Charniak-Lease parser scored 98.0 and the Bikel parser scored 97.0, averaged across both agent and target relations. They also report F_*dep *_scores of 83.5 and 83.0 for prepositional modification of nouns and verbs respectively, which are slightly better than our best parsers' scores on this task; their system contains a module specifically written to correct ambiguous prepositional phrase attachments. (Note that the F_*dep *_scores reported here are calculated from the individual precision and recall scores given in the original Schneider *et al*. paper.)

One factor common to the Pyysalo *et al*. paper and the Schneider *et al*. paper is the small size of the evaluation datasets (300 and 100 sentences respectively) since both required the manual preparation of a dependency corpus tailored to the parsers under inspection. Another advantage of producing dependency parses from constituent parses is that we can make use of the larger and rapidly-growing body of treebank-annotated biological text. Since this project was begun, the GENIA treebank has grown from 200 to 500 MEDLINE abstracts, and the BioIE project [14] has released 642 abstracts annotated in a similar format. The Stanford algorithm provides a *de facto *standard for comparing a variety of constituent parsers and treebanks at the dependency level; if the dependency parser community were to adopt the same set of grammatical relations as standard, then native dependency parsers could be compared to constituent parsers and to biological treebanks fairly and transparently.

The use of dependency graph analysis as an evaluation tool is not a new idea, having been discussed by the NLP community for several years, but to the best of our knowledge the application of such methods to specific problem domains like bioinformatics is a recent development. An early proposal along these lines [46] also acknowledged that inconsequential differences exist between different dependency representations of the same text, and included some suggested ways to exclude these phenomena, although without a comprehensive treatment. While such differences do exist, we believe that dependency graphs are much less prone to this problem than constituent trees. The same paper also discussed the mapping of constituent trees to dependency graphs via phrasal heads; the Stanford toolkit relies on a more sophisticated version of this process. Its author later used this approach to evaluate his own MINIPAR dependency parser [47]. Later, the EAGLE and SPARKLE projects used hierarchically-classified grammatical relations, which are comparable to the Stanford toolkit's dependency types, to evaluate parsers in several languages [48-50]. Similar scoring measures have been proposed for partial parsers [51,52] – those parsers which only return complete syntactic analyses of parts of each sentence. However, despite the well-known issues with constituent-based methods and the wealth of research on alternatives such as these, constituent precision and recall (along with supplementary information like number of crossing brackets per sentence) remain the *de facto *standard for reporting parser accuracy.

## Methods

### Preparing the corpus

We took the initial release of the GENIA treebank, which contains 200 abstracts from MEDLINE matching the query terms *human, blood cell *and *transcription factor*, corrected several minor errors, and removed a small number of truncated sentences. This left us with 45406 tokens (words and punctuation symbols) in 1757 sentences, from which we stripped all annotations.

Before parsing, the words in the corpus needed to be assigned POS tags. We did not use the gold standard POS tags as this would not reflect the typical use case for a parser, where the text is completely unseen. The Charniak and Charniak-Lease parsers perform POS-tagging internally, but the difference between them is that while the original Charniak parser learns to POS-tag as part of the parsing engine's training process – and therefore is distributed with a general-English POS-tagging vocabulary learnt from the Penn Treebank – the Charniak-Lease parser has a decoupled POS-tagging module which can be trained separately, and is provided pre-trained on a different part of the GENIA corpus from that which is included in the GENIA treebank. (Note that it still uses lexical statistics learnt from the Penn Treebank for the actual syntactic parsing step as there is not yet sufficient syntactically-annotated biological text for retraining the parsing engine.) The other parsers in the experiment expect pre-tagged text, for which we used the MedPost tagger [53] which is trained on text from a variety of MEDLINE abstracts.

### Parsing the corpus

All the parsers were invoked with default compile-time and command-line options, with the exception that all resource limits were set to their most generous levels to allow for particularly long/complex sentences. Some post-processing was required to normalise punctuation symbols and deal with other formatting issues, and to insert 'dummy' trees with no nesting each time one of the parsers completely failed to process a sentence. Prior to scoring, some additional operations were carried out on both the gold standard treebank and the parser output files. PRT labels were replaced with ADVP, and NAC and NX labels were replaced with NP, as these constituent types are not used in GENIA. Any constituents with a single daughter of the same type were removed, as were all constituents that did not cover any words in the sentence, and TOP nodes (S1 nodes in the case of the Charniak parser) which are meaningless top-level container constituents inserted by the parsers at the root of every sentence as a processing convenience.

The Penn Treebank defines a set of grammatical function suffixes on constituents, such as -LGS for logical subset, -LOC for location and -TMP for temporal modifier, that allow certain aspects of meaning to be represented more specifically than a purely syntactic annotation allows. GENIA uses a subset of these suffixes, the Stanford parser can generate a different subset, and the dependency graph generation algorithm can use another subset to provide additional clues for identifying the correct dependency to hold between two words. However, since these subsets do not match, and the other parsers in the evaluation do not produce any function suffixes at all, we completely discarded them in order to maintain a level playing field. There is a tool which adds these suffixes probabilistically to raw trees [29], but it was designed for the Charniak parser and is very sensitive to small differences in output between different parsers; its performance on biological text is untested so far and this would make an interesting experiment.

### Generating the dependency graphs

We will not discuss in detail the system for mapping from phrase structure trees to dependency graphs as it is described thoroughly in [9] and in the documentation for the Stanford NLP tools [54]. Briefly, it defines a taxonomy of directed, labelled grammatical relations, from the most general default type, DEPENDENT, to highly specific types such as NOMINAL_PASSIVE_SUBJECT or PHRASAL_VERB_PARTICLE. Each type has a list of allowable source constituents, target constituents and local tree structures that may hold between source and target; these definitions can include both structural constraints and lexical constraints (e.g. lists of valid words within the constituents). The algorithm attempts to match the patterns against the supplied tree structure of a sentence, from most specific to most general, and when a match is found, a dependency arc is added to the output graph from the head word of the source constituent to the head word of the target constituent. (A head word of a constituent is the word that is central to that constituent's meaning, upon which all the other words within it ultimately depend; e.g. the head of a verb phrase is the verb itself, and the head of a noun phrase is the rightmost noun.)

The algorithm also provides the facility to 'collapse' graphs into a slightly simplified form, replacing certain words such as prepositions or possessives with dependencies, and optionally adding extra dependencies that make the semantics of each sentence slightly more explicit (at the expense of making the sentence's graph potentially cyclic rather than guaranteed acyclic). When scoring the parsers' overall performance, we used the collapsed versions of the dependency graphs with all additional dependencies added in, as this is the kind of graph one would find most useful in an information extraction project. The specific subtasks for the Charniak-Lease and Bikel parsers however used the unmodified graphs as these allowed a more fine-grained analysis of behaviour.

### Scoring the parsers

The effectiveness scores F_*const *_and F_*dep *_are constituent tree and dependency graph similarity measures, respectively. They are the harmonic mean of the Precision (P) and Recall (R) values achieved by each parser, and are thus designed to penalise parsers who favour one at the expense of the other:

F=2×P×RP+R
 MathType@MTEF@5@5@+=feaafiart1ev1aaatCvAUfKttLearuWrP9MDH5MBPbIqV92AaeXatLxBI9gBaebbnrfifHhDYfgasaacH8akY=wiFfYdH8Gipec8Eeeu0xXdbba9frFj0=OqFfea0dXdd9vqai=hGuQ8kuc9pgc9s8qqaq=dirpe0xb9q8qiLsFr0=vr0=vr0dc8meaabaqaciaacaGaaeqabaqabeGadaaakeaacqWGgbGrcqGH9aqpdaWcaaqaaiabikdaYiabgEna0kabdcfaqjabgEna0kabdkfasbqaaiabdcfaqjabgUcaRiabdkfasbaaaaa@3985@

Precision is the proportion of constituents or dependents in the parsed corpus that are actually present in the gold standard:

P=#true positives#true positives+#false positives
 MathType@MTEF@5@5@+=feaafiart1ev1aaatCvAUfKttLearuWrP9MDH5MBPbIqV92AaeXatLxBI9gBaebbnrfifHhDYfgasaacH8akY=wiFfYdH8Gipec8Eeeu0xXdbba9frFj0=OqFfea0dXdd9vqai=hGuQ8kuc9pgc9s8qqaq=dirpe0xb9q8qiLsFr0=vr0=vr0dc8meaabaqaciaacaGaaeqabaqabeGadaaakeaacqWGqbaucqGH9aqpdaWcaaqaaiabcocaJiabbsha0jabbkhaYjabbwha1jabbwgaLjabbccaGiabbchaWjabb+gaVjabbohaZjabbMgaPjabbsha0jabbMgaPjabbAha2jabbwgaLjabbohaZbqaaiabcocaJiabbsha0jabbkhaYjabbwha1jabbwgaLjabbccaGiabbchaWjabb+gaVjabbohaZjabbMgaPjabbsha0jabbMgaPjabbAha2jabbwgaLjabbohaZjabgUcaRiabcocaJiabbAgaMjabbggaHjabbYgaSjabbohaZjabbwgaLjabbccaGiabbchaWjabb+gaVjabbohaZjabbMgaPjabbsha0jabbMgaPjabbAha2jabbwgaLjabbohaZbaaaaa@6C1E@

Recall is the proportion of constituents or dependents in the gold standard corpus that are correctly proposed by the parser:

R=#true positives#true positives+#false negatives
 MathType@MTEF@5@5@+=feaafiart1ev1aaatCvAUfKttLearuWrP9MDH5MBPbIqV92AaeXatLxBI9gBaebbnrfifHhDYfgasaacH8akY=wiFfYdH8Gipec8Eeeu0xXdbba9frFj0=OqFfea0dXdd9vqai=hGuQ8kuc9pgc9s8qqaq=dirpe0xb9q8qiLsFr0=vr0=vr0dc8meaabaqaciaacaGaaeqabaqabeGadaaakeaacqWGsbGucqGH9aqpdaWcaaqaaiabcocaJiabbsha0jabbkhaYjabbwha1jabbwgaLjabbccaGiabbchaWjabb+gaVjabbohaZjabbMgaPjabbsha0jabbMgaPjabbAha2jabbwgaLjabbohaZbqaaiabcocaJiabbsha0jabbkhaYjabbwha1jabbwgaLjabbccaGiabbchaWjabb+gaVjabbohaZjabbMgaPjabbsha0jabbMgaPjabbAha2jabbwgaLjabbohaZjabgUcaRiabcocaJiabbAgaMjabbggaHjabbYgaSjabbohaZjabbwgaLjabbccaGiabb6gaUjabbwgaLjabbEgaNjabbggaHjabbsha0jabbMgaPjabbAha2jabbwgaLjabbohaZbaaaaa@6BE2@

When calculating *F*_*const*_, a constituent is treated as a true positive only if its label (constituent type) and span (the portion of the sentence covered by the constituent, not counting punctuation) are correct. When calculating F_*dep*_, a dependency arc is treated as a true positive only if its label (dependency type), start node and end node are correct (unless the loose matching criterion is specified, in which case the label is disregarded).

For brevity, individual precision and recall scores have not been reported in this study. In constituent terms, and considering successfully parsed sentences only, all parsers scored slightly higher on precision than they did on recall, indicating that they were producing somewhat sparser trees than the GENIA annotators. In dependency terms, on the other hand, all parsers scored almost exactly the same for precision and recall on successfully parsed sentences. This suggests that omitted dependencies were usually replaced with a single erroneous arc.

## List of abbreviations

**F **Parser effectiveness (F-measure)

**F**_*const *_Effectiveness based on constituents

**F**_*dep *_Effectiveness based on dependencies

**NLP **Natural language processing

**P **Precision

**POS **Part of speech

**PTB **Penn Treebank

**R **Recall

The following list covers the linguistic abbreviations used in phrase-structure tree diagrams in this paper only. See [10] for explanations of their names and a comprehensive list.

**ADVP **Adverbial phrase

**CC **Coordinating conjunction

**CD **Cardinal number

**DT **Determiner

**IN **Preposition or subordinating conjunction

**NN **Noun, singular or mass

**NNS **Noun, plural

**NP **Noun phrase

**PP **Prepositional phrase

**RB **Adverb

**S **Simple declarative clause

**VBD **Verb, past tense

**VBN **Verb, past participle

**VBP **Verb, non-3rd person singular present

**VP **Verb phrase

**WDT ***Wh*-determiner (e.g. "which", "that", "whatever")

**WHNP ***Wh-noun *phrase (noun is replaced by "which", "who" etc.)

The following list covers the linguistic abbreviations used in dependency graph diagrams in this paper only. See [9] and [54] for comprehensive lists.

**ADVMOD **Adverbial modifier

**AND **Conjunction 'and'

**AUX **Auxiliary

**AUXPASS **Passive auxiliary

**BY **Preposition 'by'

**DEP **Dependent

**DET **Determiner

**DOBJ **Direct object

**DURING **Preposition 'during'

**NN **Noun compound modifier

**NSUBJ **Nominal subject

**NSUBJPASS **Passive nominal subject

**NUM **Numeric modifier

**OF **Preposition 'of'

**IN **Preposition 'in'

**RCMOD **Relative clause modifier

## Authors' contributions

ABC designed and wrote the Perl scripts and Java classes used in the experiment, analysed the results and drafted the manuscript. AJS participated in the experimental design and data analysis, and co-edited the manuscript. Both authors were involved in planning the study, and both read and approved the final manuscript.
